# The impact of electronic versus paper-based data capture on data collection logistics and on missing scores in thyroid cancer patients

**DOI:** 10.1007/s12020-023-03628-9

**Published:** 2023-12-16

**Authors:** Susanne Singer, Gerasimos Sykiotis, Akram Al-Ibraheem, Monica Pinto, Ioannis Iakovou, Arild Andre Østhus, Eva Hammerlid, Laura Deborah Locati, Eva Maria Gamper, Juan Ignacio Arraras, Susan Jordan, Matthias Buettner, Deborah Engesser, Katherine Taylor, Rita Canotilho, Georgios Ioannidis, Olga Husson, Ricardo Ribeiro Gama, Giuseppe Fanetti, Laura Moss, Johanna Inhestern, Guy Andry, Harald Rimmele, Naomi Kiyota

**Affiliations:** 1 Institute of Medical Biostatistics, Epidemiology and Informatics (IMBEI), University Medical Centre Mainz, Mainz, Germany; 2 University Cancer Centre, Mainz, Germany; 3 Service of Endocrinology, Diabetology and Metabolism, Lausanne University Hospital and University of Lausanne, Lausanne, Switzerland; 4 Department of Nuclear Medicine, King Hussein Cancer Center, Amman, Jordan; 5 Rehabilitation Medicine Unit, Strategic Health Services Department, Istituto Nazionale Tumori – IRCCS – Fondazione G. Pascale, Naples, Italy; 6 Department of Nuclear Medicine, Aristotle University, Thessaloniki, Greece; 7 ENT and Head and Neck Department, University Medical Centre Oslo, Oslo, Norway; 8 Institute of Clinical Sciences, Sahlgrenska Academy, University of Gothenburg, Gothenburg, Sweden; 9 Department of Otorhinolaryngology-Head and Neck Surgery, Sahlgrenska University Hospital, Gothenburg, Sweden; 10 Head and Neck Medical Oncology Unit, Fondazione IRCCS istituto Nazionale dei Tumori, Milan, Italy; 11 Department of Nuclear Medicine and Department of Psychiatry, Psychotherapy and Psychosomatic Medicine, University Hospital Psychiatry II, Medical University of Innsbruck, Innsbruck, Austria; 12 Oncology Departments, Complejo Hospitalario de Navarra, Pamplona, Spain; 13 School of Public Health, The University of Queensland, Brisbane, QLD Australia; 14 Instituto Português do Oncologia do Porto Francisco Gentil, Porto, Portugal; 15 Oncology Department, Nicosia General Hospital, Nicosia, Cyprus; 16 Division of Psychosocial Research and Epidemiology, Netherlands Cancer Institute, Amsterdam, The Netherlands; 17 Head and Neck Surgery Department, Barretos Cancer Hospital, Barretos, Brazil; 18 Division of Radiotherapy, Centro di Riferimento Oncologico di Aviano (CRO) IRCCS, Aviano, PN Italy; 19 Velindre Cancer Centre, Velindre University NHS Trust, Cardiff, UK; 20 Department of Otorhinolaryngology, Oberhavelkliniken, Hennigsdorf, Germany; 21 Surgery Department, Jules Bordet Institute, Brussels, Belgium; 22 Bundesverband Schilddrüsenkrebs – Ohne Schilddrüse leben e. V., Berlin, Germany; 23 Department of Medical Oncology and Hematology, Cancer Center, Kobe University Hospital, Kobe, Japan

**Keywords:** Electronic patient-reported outcomes, Mode of administration, Paper–pencil, Speed, Missing scores, Help needed

## Abstract

**Purpose:**

The purpose of this study was to investigate the impact of the type of data capture on the time and help needed for collecting patient-reported outcomes as well as on the proportion of missing scores.

**Methods:**

In a multinational prospective study, thyroid cancer patients from 17 countries completed a validated questionnaire measuring quality of life. Electronic data capture was compared to the paper-based approach using multivariate logistic regression.

**Results:**

A total of 437 patients were included, of whom 13% used electronic data capture. The relation between data capture and time needed was modified by the emotional functioning of the patients. Those with clinical impairments in that respect needed more time to complete the questionnaire when they used electronic data capture compared to paper and pencil (OR_adj_ 24.0; *p* = 0.006). This was not the case when patients had sub-threshold emotional problems (OR_adj_ 1.9; *p* = 0.48). The odds of having the researcher reading the questions out (instead of the patient doing this themselves) (OR_adj_ 0.1; *p* = 0.01) and of needing any help (OR_adj_ 0.1; *p* = 0.01) were lower when electronic data capture was used. The proportion of missing scores was equivalent in both groups (OR_adj_ 0.4, *p* = 0.42).

**Conclusions:**

The advantages of electronic data capture, such as real-time assessment and fewer data entry errors, may come at the price of more time required for data collection when the patients have mental health problems. As this is not uncommon in thyroid cancer, researchers need to choose the type of data capture wisely for their particular research question.

## Background

Assessing patient-reported outcomes (PRO) has become a standard now both in clinical trials and routine care worldwide [1, 2]; it is also relevant many years after the diagnosis [3]. In a busy clinic or in multinational trials, using electronic data capture instead of paper is often preferred for several reasons: real-time assessment and consequently immediate feedback on the patients’ well-being [4], easier logistics once the system is established [5], the possibility for the adaptive presentation of questions [6], avoidance of secondary data entry errors [7], and ease of using different language versions.

However, there are also disadvantages of this mode of data capture: costs and time involved to develop and set up the information technology (IT) infrastructure [8], increased workload for the health care professionals [9, 10], challenges with data security [11] or digital exclusion [12]. There are also reports that electronic assessments may be more challenging for some patients [13], although other studies found that most patients prefer electronic symptom monitoring [14, 15]. Generally, it seems that patients agree to report on their symptoms electronically but they want to talk to a person about their problems as soon as there is something of concern [16, 17]. Preferences and participation are also related to the patients’ age, education, and digital literacy [9, 18, 19].

Another much-debated question is whether electronic data capture versus paper and pen or a life telephone interview is equivalent in terms of the outcomes obtained. While most studies find that equivalence is sufficient [20–27], others have reported that this is not always the case [28–31]. For example, a randomized trial found that patients reported more severe problems with an automated voice response system compared to during a nurse-led live telephone interview [30].

There is conflicting evidence whether the mode of assessment affects the proportion of missing data. While some studies report better completion with electronic data capture [32, 33], others found the opposite [34]. Forced answer options usually result in no missing items at all; participants might, however, just stop with questionnaire completion.

Regarding the time needed to complete a questionnaire, evidence is also inconclusive. In a systematic review, only two out of nine studies found that the completion time was shorter with electronic devices [35]. This question, however, depends on what time is taken into account, just the time needed for filling out the questionnaire or also the time a tablet takes to start or an online version to load.

Due to these open questions, it is generally recommended to avoid mixing the modes of data capture in a given study, unless there is evidence indicating that, for the particular instrument and patient population, the discrepancies are minor [7, 36]. The aim of the present analysis was to investigate whether there were differences in time required, or the need for assistance in completing a newly developed questionnaire measuring quality of life in thyroid cancer patients [37–39]. We also examined the proportion of missing data by mode of data capture. In particular, our questions were:

1. Is the type of data capture (electronic vs. paper-based) associated with the time required to complete the questionnaire?
2. Do patients more frequently complete the questionnaire themselves (as opposed to the researcher asking the questions and entering the data/completing the forms on behalf of the patient) when they use electronic rather than paper-and-pen data capture?
3. Is the type of data capture (electronic vs. paper-based) associated with whether or not help is required to complete the questionnaire?
4. Does the proportion of items missing a response vary by type of data capture?

## Methods

### Study design

We used data from the phase IV field validation study of the European Organisation for Research and Treatment of Cancer (EORTC) Quality of Life Questionnaire Thyroid Module (EORTC QLQ-THY34; study number EORTC 002/2017), to address our research questions. This was a prospective multinational study including patients with thyroid cancer from 21 different institutions in 17 countries.

Two groups of patients were enrolled. The first group comprised patients about to undergo treatment, named Group 1 (treatment). They completed the questionnaire on three occasions: before onset of treatment or best supportive care (t1) as well as 6 weeks (t2) and 6 months thereafter (t3). If a collaborating institution was not able to include patients at t1, patients could be enrolled at t2 (then defined as 6 weeks after first day of initial treatment). The second group comprised people who had been diagnosed with thyroid cancer ≥24 months prior to enrollment, without structural evidence of disease based on imaging^1^, and without anti-neoplastic treatment during the past 12 months, named Group 2 (survivors). They completed the questionnaire only once.^2^ As there is no generally accepted definition of when a patient could be considered to be a survivor, we chose the 24-month time interval, together with the other criteria, assuming that the participants would then not be in an acute situation anymore.

Eligibility criteria for all participants were: diagnosed thyroid cancer, sufficient language proficiency (the questionnaire was available in the local language of the participating institutions) and cognitive functioning to understand and complete the forms (as judged by the local investigator), age ≥16 years, and written informed consent.

Institutional Review Board Approval was obtained from the Ethics Committee of Rhineland-Palatinate Medical Association with reference number 837.406.17 (11240) as well as from all participating sites, according to the respective national requirements. More details about the study design and conduct are published elsewhere [37].

### Mode of questionnaire administration and data capture

Electronic data capture was undertaken using the Computer Based Health Evaluation System (CHES) [40, 41]. Each site was free to decide what type of data capture (paper vs. electronic) they used, depending on the local infrastructure.

At the first time-point (t1), all patients were seen in person as per the study protocol. Electronic data capture could be used by handing them a tablet or using an online system.

### Instruments

The participants received two questionnaires: the core instrument of the EORTC, the EORTC QLQ-C30 [42], and the thyroid cancer-specific module, the EORTC QLQ-THY34 [37].

The thresholds published by Giesinger et al. [43] were used to define what patients had clinically relevant impairments of their subjective emotional and cognitive functioning, which are 71 and 75, respectively.

The local investigator asked a few debriefing questions at t1 regarding the time needed to complete the questionnaire (core questionnaire and module together), type of completion (self-completed vs. researcher read questions to participants and recorded their response), and whether any help was required to complete it (no help, practical help [for example when the patient did not have their glasses so the questionnaire had to be read to them, or they had shaky hands and had difficulty writing], supportive help [such as interviewer just sitting with the patient while they completed the questionnaire], or help with understanding the questionnaire).

### Statistical analysis

Data analysis comprising descriptive statistics and multivariable binary logistic regression analyses was performed with STATA (StataCorp. 2017. Stata Statistical Software: Release 16. College Station, TX: StataCorp LP).

The exposure variable was the type of data capture (paper vs. CHES) and the outcomes were: time needed (<10 vs. ≥10 min), type of completion (self-completed vs. orally), help required (any help vs. no help), and proportion of missing scale scores. According to the EORTC scoring manual [44], a score of a certain scale can be calculated when at least half of the items of that scale are completed. Hence, if more than half of the items are not completed, no score is computed and is therefore missing. We first calculated the number of missing scores per participant and then created a binary variable “any missing score vs. no missing score”. The latter was used as an outcome variable.

We explored the potential for effect modification by the following variables: age (<75 vs. ≥75 years), education (<10 years, 10 years, >10 years), clinical impairment of cognitive or emotional functioning at baseline; effect modification was explored using the Mantel-Haenszel method and consequently tested with likelihood ratio tests in the regression models. If there was no evidence for effect modification, we did not specifically mention that in the following results section.

The following variables were adjusted for language, UICC stage, performance status, comorbidity (ascertained using the Charlson Comorbidity Score), exhaustion at t1 (measured with the EORTC QLQ-THY34). We did not adjust for further individual patient characteristics because the type of data capture did not vary within one center, for logistic reasons. Once the center had decided about the type of data capture, this was not changed later on. For that reason, the results are not confounded by this.

As patients with anaplastic thyroid cancer and those receiving best supportive care differ in certain clinical aspects from those with other histologies and treatments, we explored in a sensitivity analysis whether the effect of data capture on the various outcomes is different in these patients.

## Results

### Sample characteristics

**Fig. 1:**
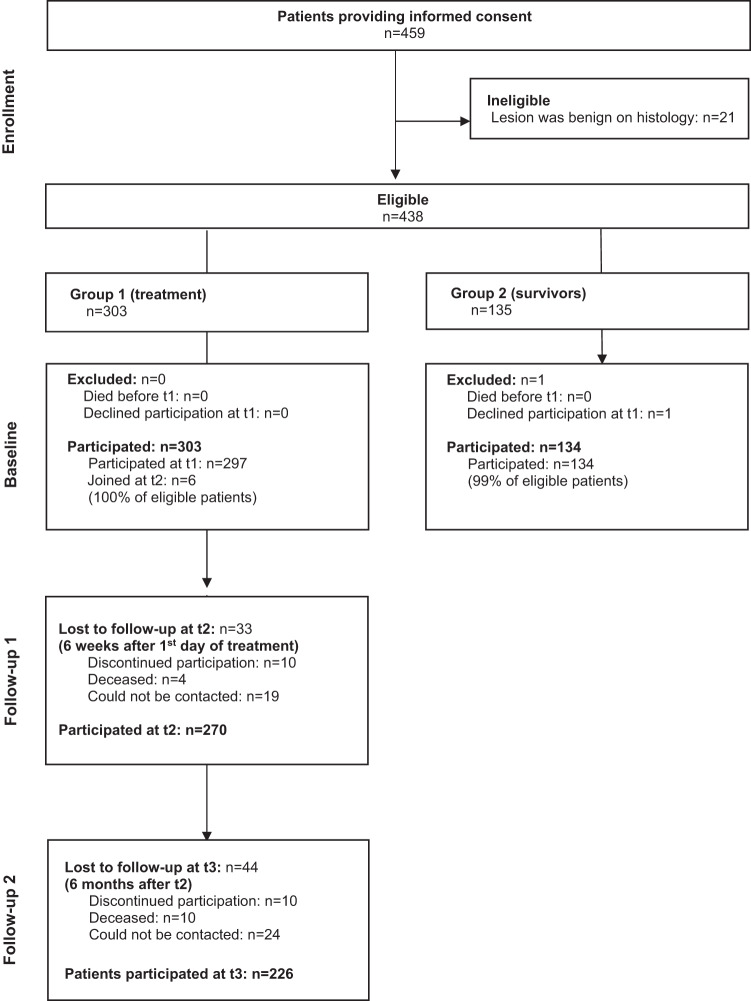
Patient flow through the study

A total of 437 patients participated (see Fig. 1 for details). The majority (84%) had differentiated thyroid cancer, 11% had medullary, 4% anaplastic, and 1% other types of thyroid cancer. About 9% were 75 years or older. The median age was 51 years (mean: 51 years, standard deviation 16). Most (71%) had received more than 10 years of education (Table 1). At the time of entry into the study, 278 (64%) participants had received total thyroidectomy, 35 (8%) partial thyroidectomy, 72 (16%) no surgery, and for 52 (12%) no information about surgery was available. By the end of the study, 364 (83%) had received total thyroidectomy, 44 (10%) partial thyroidectomy, and 29 (7%) no surgery.

**Table 1:** Demographic and clinical characteristics of the participants by type of data capture

	Total (*n* = 437)	Paper (*n* = 383)	Electronic (*n* = 54)	*p* value
	*N* (%)	*N* (%)	*N* (%)	
Sex				
Male	118 (27%)	107 (28%)	11 (20%)	0.24
Female	319 (73%)	276 (72%)	43 (80%)	
Age				
<75 years	131 (30%)	351 (92%)	49 (91%)	0.82
≥75 years	81 (19%)	32 (8%)	5 (9%)	
Education				
<10 years	68 (16%)	56 (15%)	12 (22%)	0.25
10 years	45 (10%)	41 (11%)	4 (7%)	
>10 years	311 (71%)	273 (71%)	38 (70%)	
Missing information	13 (3%)	13 (3%)	0 (0%)	
Histology				
Papillary	304 (70%)	275 (72%)	29 (54%)	<0.001
Follicular	45 (10%)	28 (7%)	17 (31%)	
Hurthle-cell	10 (2%)	10 (3%)	0 (0%)	
Poorly differentiated	7 (2%)	7 (2%)	0 (0%)	
Medullary	47 (11%)	42 (11%)	5 (9%)	
Anaplastic	19 (4%)	16 (4%)	3 (6%)	
Other	5 (1%)	5 (1%)	0 (0%)	
UICC^a^				
UICC I	202 (46%)	219 (57%)	26 (48%)	0.19
UICC II	57 (13%)	52 (14%)	7 (13%)	
UICC III	31 (7%)	29 (8%)	2 (4%)	
UICC IV	68 (16%)	54 (14%)	14 (26%)	
Unknown	79 (18%)	29 (8%)	5 (9%)	
Current status of disease^b^				
No evidence of disease	143 (33%)	130 (34%)	13 (24%)	0.001
Indeterminate	22 (5%)	13 (3%)	9 (17%)	
Biochemically incomplete	21 (5%)	18 (5%)	3 (6%)	
Structural disease	160 (37%)	143 (37%)	17 (31%)	
Unknown to the collaborator	84 (19%)	72 (19%)	12 (22%)	
Missing information	7 (2%)	7 (2%)	0 (0%)	
Karnofsky performance status				
Mean (SD)	92 (11)	92 (11)	93 (11)	0.69
Charlson comorbidity score				
Mean (SD)	1 (2)	1 (2)	2 (2)	<0.001

^a^Usually taken from t2. If not available, then t1 or t3

^b^At t

Before t1, 10 (2%) patients had had radiotherapy for local control and 8 for distant metastases; 10 (2%) had received tyrosine kinase inhibitors (TKI); radioactive iodine (RAI) was received by 36 (8%) for ablation and 69 (16%) for therapy. By the end of the study, 275 (63%) participants had ever received RAI and 36 (8%) TKI. There were 4 patients who received the best supportive care.

Clinically relevant impairment of cognitive and emotional functioning as defined by Giesinger et al. (2020) was present at baseline in 36% and 50%, respectively (Table 2).

**Table 2:** Aspects of quality of life by type of data capture

	Total (*n* = 437)	Paper (*n* = 383)	Electronic (*n* = 54)	*p* value
	*N* (%)	*N* (%)	*N* (%)	
Cognitive functioning				
Below TCI	275 (63%)	244 (64%)	31 (57%)	0.34
Clinically important impairment	155 (35%)	132 (34%)	23 (43%)	
Unknown	7 (2%)	7 (2%)	0 (0%)	
Emotional functioning				
Below TCI	216 (49%)	190 (50%)	26 (48%)	0.57
Clinically important impairment	214 (49%)	186 (49%)	28 (52%)	
Unknown	7 (2%)	7 (2%)	0 (0%)	
Exhaustion				
Mean (SD)	33 (28)	33 (28)	30 (24)	0.40

*TCI* threshold for clinical impairment [43]

### Type of data capture

A total of 54 (12%) participants from three sites (Kobe, Japan; Innsbruck, Austria; Pamplona, Spain) used CHES for electronic data capture. The remaining data were captured on paper.

### Description of outcomes

#### Time required

About 42% of the participants needed <10 min and 55% needed ≥10 min. For 12 participants (3%), the time required to complete the questionnaire was not documented. The numbers and percentages broken down by type of data capture are displayed in Table 3.

**Table 3:** Time and help required as well as proportion of missing scores by type of data capture

	Total (*n* = 437)	Paper (*n* = 383)	Electronic (*n* = 54)	*p* value
	*N* (%)	*N* (%)	*N* (%)	
Time required				
<10 min	183 (42%)	170 (44%)	13 (24%)	0.01
≥10 min	242 (55%)	204 (53%)	38 (70%)	
Not documented	12 (3%)	9 (2%)	3 (6%)	
Type of completion				
Self-completed	298 (68%)	254 (66%)	44 (81%)	0.07
Researcher read the questions	130 (30%)	120 (31%)	10 (19%)	
Not documented	9 (2%)	9 (2%)	0 (0%)	
Help needed				
No help required	323 (74%)	279 (73%)	44 (81%)	0.30
Any help required	106 (24%)	96 (25%)	10 (19%)	
Not documented	8 (2%)	8 (2%)	0 (0%)	
Missing scales scores				
No missing scores	421 (96%)	368 (96%)	53 (98%)	0.45
At least 1 missing score	16 (4%)	15 (4%)	1 (2%)	

#### Type of completion

The questionnaires were self-completed by 68% of participants, by the researcher in 30%, and in 2% this was not documented (Table 3).

The type of completion was associated with the language used. The proportion of oral completion (researcher read out the questions) was as follows: Swedish 75%, Greek 61%, Portuguese 53%, French 30%, Spanish 30%, Italian 28%, German 28%, Japanese 27%, English 7%, Dutch 7%, Norwegian 4%, Arabic 2% (*p* < 0.01).

#### Help needed

The majority (74%) of the participants required no help to complete the questionnaires. A quarter (24%) needed some type of help, and for 2% whether help was needed was not documented (Table 3).

#### Proportion of missing scale scores

At t1, there were 16 participants (4% of those participating at t1) with missing scale scores; in 13 of these cases, only 1 score was missing.

At t2, there were 10 participants (3% of those participating at t2) with missing scale scores; in 8 of them, only 1 score was missing.

At t3, there were 14 participants (6% of those participating at t3) with missing scale scores; in 11 of them, only 1 score was missing.

For the 6 participants who entered the study at t2, their completion data were used from t2 for the following analyses. They all had no missing scores at t2. Hence, the final number for the regression models was 16 (4%) with at least one missing scale score.

### Association of type of data capture with time or help required and with missing scores

#### Is the type of data capture associated with the time data collection needs?

There was a social gradient with less time needed the higher the education level was (≥10 min needed in participants with less than 10 years versus 10 years versus more than 10 years of schooling: 76% vs. 60% vs. 53%), but effect modification was not observed. A similar pattern was seen regarding cognitive functioning. Emotional functioning, however, did modify the effect of data capture on time needed. Consequently, stratum-specific effect estimates for patients with and without clinical impairment of emotional functioning are reported in the following, and the other variables were treated as potential confounders. Hence, the final list of variables adjusted for the regression model were age, education, cognitive functioning, language, UICC stage, status of disease, performance status, comorbidity, and exhaustion.

**Fig. 2:**
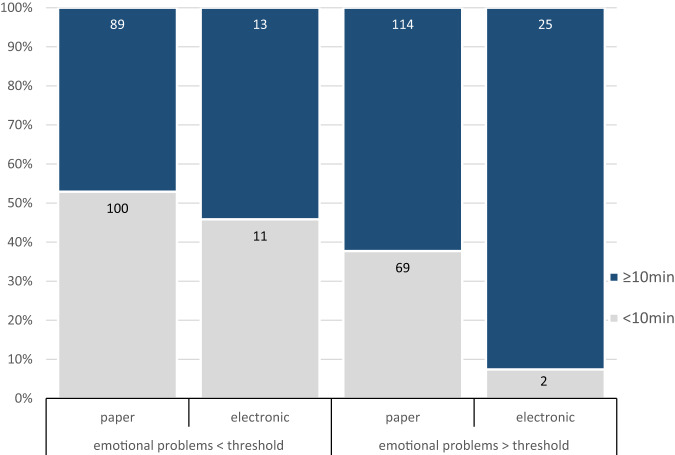
Proportion of patients needing less or more than 10 min to complete the questionnaire, by type of data capture and level of emotional problems. The figures inside the columns indicate the absolute numbers of participants within that category

With this model, there was no evidence that patients without clinically relevant emotional problems differed in the time needed to complete the questionnaires (adjusted odds ratio [OR_adj_] 1.9, 95% confidence interval [CI] 0.3–11.4; *p* = 0.48). In contrast to that, patients with clinically relevant emotional problems more often needed ≥10 min to complete the questionnaires when they used electronic data capture compared to paper and pencil (OR_adj_ 24.0, CI 2.4–235.8; *p* = 0.006; Fig. 2).

When we ignore effect modification by emotional functioning, the odds of needing more than 10 min for questionnaire completion was 8 times higher in patients who used electronic data capture compared to paper and pencil (OR_adj_ 5.5, CI 1.1–27.3; *p* = 0.04).

#### Do patients more frequently self-complete the questionnaire when they use electronic data capture?

The odds of having the researcher reading the questions out (instead of the patient doing this themselves) were considerably lower when electronic data capture was used (OR_adj_ 0.1, CI 0.03–0.6; *p* = 0.01) compared to paper-based data collection.

#### Is the type of data capture associated with the help required for data collection?

The odds of needing any help were lower when electronic data capture was used (OR_adj_ 0.1, CI 0.02–0.6; *p* = 0.01) compared to paper-based data collection.

#### Does the proportion of missing scale scores differ by type of data capture?

The proportion of missing scale scores was similar in both types of data capture: 4% vs. 2% had at least one missing scale score in paper vs. electronic data capture (see Table 3 for details). In line with that, when adjusting for potential confounders, we found no evidence for an effect of data capture on the likelihood of missing scores (OR_adj_ 0.4, CI 0.04–4.0; *p* = 0.42).

### Sensitivity analysis

#### Anaplastic cancer versus other types of histology

Of the 19 patients with anaplastic cancer, 3 had used CHES and the remaining used paper for their data capture. More than half (58%, *n* = 11) reported clinically relevant levels of emotional problems. The majority (84%, *n* = 16) needed more than 10 min for completing the questionnaires. Fifty-eight percent (*n* = 11) completed the questionnaires on their own. Ten percent (*n* = 2) had at least one missing score at t1. The anaplastic cancer patients were considerably older than the patients with other histologies (median 72 years, mean 70, range 43–83 years).

Due to the low number of patients using CHES in this group of patients, regression models using the same specifications as with the entire sample (variables taken into account for adjustment and effect modification) could not be computed. We had to restrict the adjustment variables to age and stratify by emotional functioning for the model where we had found effect modification in the entire sample. In those with increased levels of emotional problems, the age-adjusted OR for electronic data capture on time needed to complete the questionnaire was 0.13 (*p* = 0.24). For the eight patients with sub-threshold emotional problems and for all other outcomes, no regression models could be computed due to the low number of cases.

#### Best supportive care versus other treatment

All of the four patients with the best supportive care had used paper for data capture. Hence, no comparisons between different types of data capture were possible here.

## Discussion

This analysis set out to investigate the effect of electronic versus paper-based data capture on the time and help needed to complete the questionnaires for a newly developed questionnaire to measure the quality of life in thyroid cancer patients [37]. We were also interested in whether the proportion of missing scores differs between these two options.

We found that the effect of data capture on the time needed is probably modified by the emotional well-being of the patients. In those without clinically relevant mental health problems, the questionnaire completion time is similar for both types of data capture. This is in contrast to other studies where less time was needed for electronic data capture [45], though the majority of studies did not find a difference in time for completion [35]. Those exceeding the threshold for clinical importance, however, needed in our study more time when electronic data capture was used. This finding is of relevance because emotional problems are common among cancer patients; about a third suffer from co-morbid mental health conditions [46–49]. As thyroid cancer involves the endocrine system, which is related to mental health, this topic is of particular concern in this group of patients [50–56]. They often suffer from depression, anxiety and poor emotional functioning, even for long periods of time after the diagnosis [3, 57–59]. In our study, half of the participants indicated emotional problems of clinical relevance. The instrument used to capture emotional functioning, the EORTC QLQ-C30, subsumes the following constructs under it: tension, worry, irritability, and depression. Especially irritability and tension are known to be related to hyperthyroidism, while depression is more common during hypothyroid states. Thyroid cancer patients can suffer from both.

It is well-known that individuals with emotional problems may slow down in their capabilities of processing thoughts and deciding [60–63]. Recent data on digital interventions also suggest that depressive patients need professional support by a human being and should not be left alone with a tablet or computer [64]. This underlines that using the latest technology is not always the best option for data capture [24] and researchers need to think carefully about their target population when deciding about the methods of data collection, be it “paper or plastic” [23].

Apart from that particular problem, our data show that electronic data capture may be associated with needing less help in filling out the questionnaires and with a higher probability of self-completion, which is usually desired in large-scale observational studies and clinical trials. This might seem somewhat contradictory to the findings discussed above about the time needed. It might imply that the electronic data capture procedures were clear to the patients and the material was easy to use, but that some of the patients still needed more time to complete the forms electronically, for other reasons than needing help.

The proportion of missing scores was equal in both types of data capture, which is in contrast to Blondin’s study where more missing values were found with electronic data capture [34]. This difference could be explained by the different modes of data acquisition: they used an interactive voice response system, whereas we presented a questionnaire on a tablet. Moreover, they elicited daily reports from the patients, whereas our study only had three timepoints for data collection, with a longer time in between.

The results of our study should be interpreted in the light of its limitations. First of all, the type of data capture was not randomly allocated to either institutions or individual patients. The differences found could therefore also be due to residual confounding. There could also be more effect modifiers that we did not take into account. We selected only four variables for test of effect modification in order to keep the power of the tests at an acceptable level. This brings us to the next limitation which is the relatively low proportion of sites (and therefore participants) using electronic data capture. Consequently, the statistical tests used do not have the ability to detect smaller effects and effect estimates may be imprecise. Moreover, the three sites using CHES are most likely not representative for other institutions because they had a preference for this type of data capture. We also learned that one of these sites included patients at the nuclear medicine department, where internet connections were slow in some rooms due to necessary radiation protection measures there. Finally, the time required for completing the questionnaires was not measured exactly but recorded by the researchers. It is possible that it was remembered differently when using paper versus electronic data capture, thereby introducing an information bias that cannot be controlled for.

Strengths of our study include that all data collection that we considered in this analysis was done in the hospitals, so the home environment of the patients could not affect the results.

In summary, the obvious advantages of electronic data capture such as real-time assessment [4] and fewer data entry errors [7] may come at the price of additional time required for data collection on the part of the patients when they have mental health problems. As such problems are very common among thyroid cancer patients [57, 65, 66], researchers and clinicians should take these aspects into consideration when choosing a type of data capture for a particular research question or clinical application, respectively, in this patient population.

## Data Availability

The data of this study are stored in the EORTC data repository and can be accessed by other researchers.

## Footnotes

1 Survivors could have biochemically incomplete response or indeterminate response.

2 For the purpose of testing retest reliability, the questionnaire was presented a second time shortly after the first measurement, but these data were not used in the current analysis.

## References

[1] Ishiki H, Kikawa Y, Terada M, Mizusawa J, Honda M, Iwatani T, Mizutani T, Mori K, Nakamura N, Miyaji T (2023). Patient-reported outcome and quality of life research policy: Japan Clinical Oncology Group (JCOG) policy. Jpn. J. Clin. Oncol..

[2] Velikova G, Coens C, Efficace F, Greimel E, Groenvold M, Johnson C, Singer S, van de Poll-Franse L, Young T, Bottomley A (2012). Health-related quality of life in EORTC clinical trials – 30 years of progress from methodological developments to making a real impact on oncology practice. Eur. J. Cancer Suppl..

[3] Dionisi-Vici M, Fantoni M, Botto R, Nervo A, Felicetti F, Rossetto R, Gallo M, Arvat E, Torta R, Leombruni P (2021). Distress, anxiety, depression and unmet needs in thyroid cancer survivors: a longitudinal study. Endocrine.

[4] Abernethy AP, Ahmad A, Zafar SY, Wheeler JL, Reese JB, Lyerly HK (2010). Electronic patient-reported data capture as a foundation of rapid learning cancer care. Med. Care.

[5] Cramon P, Rasmussen AK, Bonnema SJ, Bjorner JB, Feldt-Rasmussen U, Groenvold M, Hegedus L, Watt T (2014). Development and implementation of PROgmatic: A clinical trial management system for pragmatic multi-centre trials, optimised for electronic data capture and patient-reported outcomes. Clin. Trials.

[6] Petersen MA, Aaronson NK, Arraras J, Chie WC, Conroy T, Costantini A, Dirven L, Fayers PM, Gamper EM, Giesinger JM (2018). The EORTC CAT Core-The computer adaptive version of the EORTC QLQ-C30 questionnaire. Eur. J. Cancer.

[7] O’Donohoe P, Reasner DS, Kovacs SM, Byrom B, Eremenco S, Barsdorf AI, Arnera V, Coons SJ (2023). Updated recommendations on evidence needed to support measurement comparability among modes of data collection for patient-reported outcome measures: a good practices report of an ISPOR Task Force. Value Health.

[8] Zebralla V, Pohle N, Singer S, Neumuth T, Dietz A, Stier-Jarmer M, Boehm A (2016). Vorstellung des Screeningsystems (OncoFunction) für Funktionsstörungen im Kopf-Hals-Tumor-follow-up. Laryngorhinootologie.

[9] Steiert C, Lambeck J, Grauvogel TD, Beck J, Grauvogel J (2023). Digital patient-reported outcome measures assessing health-related quality of life in skull base diseases-analysis of feasibility and pitfalls two years after implementation. Healthcare.

[10] T.R. Kiderlen, A. Schnack, M. de Wit Essential barriers and considerations for the implementation of electronic patient-reported outcome (ePRO) measures in oncological practice: contextualizing the results of a feasibility study with existing literature. Z. Gesundh. Wiss. 1–18 (2022) 10.1007/s10389-022-01767-3 PMC9613453 36320803

[11] Tran C, Dicker A, Leiby B, Gressen E, Williams N, Jim H (2020). Utilizing digital health to collect electronic patient-reported outcomes in prostate cancer: single-arm pilot trial. J. Med. Internet Res..

[12] J. Fischer, K. Vltavska: digital literacy and digital exclusion of poor, unemployed, uneducated and pensioners: who is the most threatened?, *27th Interdisciplinary Information Management Talks Conference (IDIMT)*. Schriftenreihe Informatik. Kutna Hora, Trauner Verlag, 2019, pp 75–82

[13] Zhu BQ, Feng TN, Izci-Balserak B (2021). Using research electronic data capture for longitudinal assessment among older adults with diabetes enhances real-time data collection. Cin. Comput. Inform. Nurs..

[14] Wintner LM, Giesinger JM, Zabernigg A, Rumpold G, Sztankay M, Oberguggenberger AS, Gamper EM, Holzner B (2015). Evaluation of electronic patient-reported outcome assessment with cancer patients in the hospital and at home. BMC Med. Inform. Decis. Mak..

[15] Velikova G, Wright EP, Smith AB, Cull A, Gould A, Forman D, Perren T, Stead M, Brown J, Selby PJ (1999). Automated collection of quality-of-life data: a comparison of paper and computer touch-screen questionnaires. J. Clin. Oncol..

[16] Alexander KE, Ogle T, Hoberg H, Linley L, Bradford N (2021). Patient preferences for using technology in communication about symptoms post hospital discharge. BMC Health Serv. Res..

[17] Olmsted SS, Grabenstein JD, Jain AK, Lurie N (2006). Patient experience with, and use of, an electronic monitoring system to assess vaccination responses. Health Expect..

[18] Graf J, Sickenberger N, Brusniak K, Matthies LM, Deutsch TM, Simoes E, Plappert C, Keilmann L, Hartkopf A, Walter CB (2022). Implementation of an electronic patient-reported outcome app for health-related quality of life in breast cancer patients: evaluation and acceptability analysis in a two-center prospective trial. J. Med. Internet Res..

[19] Kosowicz L, Tran K, Khanh TT, Dang THA, Pham V, Kim HTT, Duong HTB, Nguyen TD, Phuong AT, Le TH (2023). Lessons for Vietnam on the use of digital technologies to support patient-centered care in low- and middle-income countries in the Asia-Pacific region: scoping review. J. Med. Internet Res..

[20] Gwaltney CJ, Shields AL, Shiffman S (2008). Equivalence of electronic and paper-and-pencil administration of patient-reported outcome measures: a meta-analytic review. Value Health.

[21] Muehlhausen W, Doll H, Quadri N, Fordham B, O’Donohoe P, Dogar N, Wild DJ (2015). Equivalence of electronic and paper administration of patient-reported outcome measures: a systematic review and meta-analysis of studies conducted between 2007 and 2013. Health Qual. Life Outcomes.

[22] Romero H, DeBonis D, O’Donohoe P, Wyrwich KW, Arnera V, Platko JV, Willgoss T, Harris K, Crescioni M, Steele S (2022). Recommendations for the electronic migration and implementation of clinician-reported outcome assessments in clinical trials. Value Health.

[23] Green AS, Rafaeli E, Bolger N, Shrout PE, Reis HT (2006). Paper or plastic? Data equivalence in paper and electronic diaries. Psychol. Methods.

[24] Weiler K, Christ AM, Woodworth GG, Weiler RL, Weiler JM (2004). Quality of patient-reported outcome data captured using paper and interactive voice response diaries in an allergic rhinitis study: is electronic data capture really better?. Ann. Allergy Asthma Immunol..

[25] Rutherford C, Costa D, Mercieca-Bebber R, Rice H, Gabb L, King M (2016). Mode of administration does not cause bias in patient-reported outcome results: a meta-analysis. Qual. Life Res..

[26] Kamo N, Dandapani SV, Miksad RA, Houlihan MJ, Kaplan I, Regan M, Greenfield TK, Sanda MG (2011). Evaluation of the SCA instrument for measuring patient satisfaction with cancer care administered via paper or via the Internet. Ann. Oncol..

[27] Rasmussen SL, Rejnmark L, Ebbehoj E, Feldt-Rasmussen U, Rasmussen AK, Bjorner JB, Watt T (2016). High level of agreement between electronic and paper mode of administration of a thyroid-specific patient-reported outcome, ThyPRO. Eur. Thyroid J..

[28] Lee MK, Beebe TJ, Yost KJ, Eton DT, Novotny PJ, Dueck AC, Frost M, Sloan JA (2021). Score equivalence of paper-, tablet-, and interactive voice response system-based versions of PROMIS, PRO-CTCAE, and numerical rating scales among cancer patients. J. Patient Rep. Outcomes.

[29] Schmier JK, Kane DW, Halpern MT (2005). Practical applications of usability theory to electronic data collection for clinical trials. Contemp. Clin. Trials.

[30] Sikorskii A, Given CW, Given B, Jeon S, You M (2009). Differential symptom reporting by mode of administration of the assessment automated voice response system versus a live telephone interview. Med. Care.

[31] Juniper EF, Langlands JM, Juniper BA (2009). Patients may respond differently to paper and electronic versions of the same questionnaires. Respir. Med..

[32] Gurland B, Alves-Ferreira PC, Sobol T, Kiran RP (2010). Using technology to improve data capture and integration of patient-reported outcomes into clinical care: pilot results in a busy colorectal unit. Dis. Colon Rectum.

[33] Roick J, Danker H, Kersting A, Briest A, Dietrich A, Dietz A, Einenkel J, Papsdorf K, Lordick F, Meixensberger J (2018). Factors associated with non-participation and dropout among cancer patients in a cluster-randomised controlled trial. Eur. J. Cancer Care.

[34] Blondin JM, Abu-Hasaballah KS, Tennen H, Lalla RV (2010). Electronic versus paper diaries: a pilot study of concordance and adherence in head and neck cancer patients receiving radiation therapy. Head. Neck Oncol..

[35] Dale O, Hagen KB (2007). Despite technical problems personal digital assistants outperform pen and paper when collecting patient diary data. J. Clin. Epidemiol..

[36] Eremenco S, Coons SJ, Paty J, Coyne K, Bennett AV, McEntegart D, Force IPMMT (2014). PRO data collection in clinical trials using mixed modes: report of the ISPOR PRO mixed modes good research practices task force. Value Health.

[37] Singer S, Al-Ibraheem A, Pinto M, Iakovou I, Osthus A, Hammerlid E, Locati LD, Gamper EM, Arraras J, Jordan S (2023). International phase IV field study for the reliability and validity of the European Organisation for Research and Treatment of Cancer Thyroid Cancer Module EORTC QLQ-THY34. Thyroid.

[38] Singer S, Jordan S, Locati L, Pinto M, Tomaszewska IM, Araújo C, Hammerlid E, Vidhubala E, Husson O, Kiyota N (2017). The EORTC module for quality of life in patients with thyroid cancer: phase III. Endocr. Relat. Cancer.

[39] Singer S, Husson O, Tomaszewska IM, Locati L, Kiyota N, Scheidemann-Wesp U, Hofmeister D, Winterbotham M, Brannan C, Araújo C (2016). Quality-of-life priorities in patients with thyroid cancer: a multinational European organisation for research and treatment of cancer phase I study. Thyroid.

[40] Holzner B, Giesinger JM, Pinggera J, Zugal S, Schopf F, Oberguggenberger AS, Gamper EM, Zabernigg A, Weber B, Rumpold G (2012). The Computer-based Health Evaluation Software (CHES): a software for electronic patient-reported outcome monitoring. BMC Med. Inform. Decis. Mak..

[41] Erharter A, Giesinger J, Kemmler G, Schauer-Maurer G, Stockhammer G, Muigg A, Hutterer M, Rumpold G, Sperner-Unterweger B, Holzner B (2010). Implementation of computer-based quality-of-life monitoring in brain tumor outpatients in routine clinical practice. J. Pain. Symptom Manage..

[42] Aaronson N, Ahmedzai S, Bergmann B, Bullinger M, Cull A, Duez NJ, Filiberti A, Flechtner H, de Haes JCJM, Kaasa S (1993). The European Organization for Research and Treatment of Cancer QLQ-C30: a quality-of-life instrument for use in international clinical trials in oncology. J. Nat. Cancer Inst..

[43] Giesinger JM, Loth FLC, Aaronson NK, Arraras JI, Caocci G, Efficace F, Groenvold M, van Leeuwen M, Petersen MA, Ramage J (2020). Thresholds for clinical importance were established to improve interpretation of the EORTC QLQ-C30 in clinical practice and research. J. Clin. Epidemiol..

[44] P. Fayers, N. Aaronson, K. Bjordal, M. Groenvold, D. Curran, A. *Bottomley: EORTC QLQ-C30 Scoring Manual*, 3rd edn. (EORTC, Brüssel, 2001)

[45] Yu JY, Goldberg T, Lao NI, Feldman BM, Goh YI (2021). Electronic forms for patient reported outcome measures (PROMs) are an effective, time-efficient, and cost-minimizing alternative to paper forms. Pediatr. Rheumatol..

[46] Mitchell AJ, Chan M, Bhatti H, Halton M, Grassi L, Johansen C, Meader N (2011). Prevalence of depression, anxiety, and adjustment disorder in oncological, haematological, and palliative-care settings: a meta-analysis of 94 interview-based studies. Lancet Oncol..

[47] Singer S, Das-Munshi J, Brähler E (2010). Prevalence of mental health conditions in cancer patients in acute care – a meta-analysis. Ann. Oncol..

[48] Akechi T, Okuyama T, Sugawara Y, Nakano T, Shima Y, Uchitomi Y (2004). Major depression, adjustment disorders, and post-traumatic stress disorder in terminally ill cancer patients: associated and predictive factors. J. Clin. Oncol..

[49] Singer S, Szalai C, Briest S, Brown A, Dietz A, Einenkel J, Jonas S, Konnopka A, Papsdorf K, Langanke D (2013). Co-morbid mental health conditions in cancer patients at working age – prevalence, risk profiles, and care uptake. Psycho. Oncol..

[50] Himmerich H, Fulda S, Linseisen J, Seiler H, Wolfram G, Himmerich S, Gedrich K, Kloiber S, Lucae S, Ising M (2008). Depression, comorbidities and the TNF-alpha system. Eur. Psychiatry.

[51] Tagay S, Herpertz S, Langkafel M, Erim Y, Freudenberg L, Schopper N, Bockisch A, Senf W, Gorges R (2005). Health-related quality of life, anxiety and depression in thyroid cancer patients under short-term hypothyroidism and TSH-suppressive levothyroxine treatment. Eur. J. Endocrinol..

[52] Larisch R, Kley K, Nikolaus S, Sitte W, Franz M, Hautzel H, Tress W, Muller HW (2004). Depression and anxiety in different thyroid function states. Horm. Metab. Res..

[53] Büttner M, Krogh D, Siggelkow H, Singer S (2022). What are predictors of impaired quality of life in patients with hypoparathyroidism? – Results of a patient survey. Clin. Endocrinol..

[54] Büttner M, Krogh D, Siggelkow H, Singer S (2023). Impairments in quality of life and predictors of symptom burden in patients with hypoparathyroidism: results from a population-based survey. Endocrine.

[55] Monzani ML, Piccinini F, Boselli G, Corleto R, Margiotta G, Peeters RP, Simoni M, Brigante G (2023). Changes in quality of life after thyroidectomy in subjects with thyroid cancer in relation to the dose of levothyroxine. J. Endocrinol. Investig..

[56] W.L. Chan, H.C.W. Choi, B. Lang, K.P. Wong, K.K. Yuen, K.O. Lam, V.H.F. Lee, D. Kwong. Health-related quality of life in asian differentiated thyroid cancer survivors. Cancer Control. **28**, 10732748211029726 (2021) 10.1177/10732748211029726 PMC8252343 34189945

[57] Singer S, Lincke T, Gamper E, Schreiber S, Hinz A, Bhaskaran K, Schulte T (2012). Quality of life in patients with thyroid cancer compared with the general population. Thyroid.

[58] Gamper EM, Wintner LM, Rodrigues M, Buxbaum S, Nilica B, Singer S, Giesinger JM, Holzner B, Virgolini I (2015). Persistent quality of life impairments in differentiated thyroid cancer patients: results from a monitoring program. Eur. J. Nucl. Med. Mol. Imaging.

[59] Yang SJ, Xu XQ (2022). Anxiety and quality of life among papillary thyroid cancer patients awaiting final pathology results after surgery. Endocrine.

[60] Yudilevich P, BenEliahu E (2022). Bion’s alpha-function as a key for understanding emotional problems in ADHD. Psychoanal. Psychol..

[61] Schneider BC, Diedrich S, Hauschildt M, Biedermann SV, Arlt S, Moritz S, Jelinek L (2021). Changes in processing speed, cognitive flexibility, and selective attention over a four-week treatment period in inpatients with moderate to severe depression. Z. Fur Neuropsychologie..

[62] Mougias A, Christidi F, Synetou M, Kotrotsou I, Valkimadi P, Politis A (2019). Differential effect of demographics, processing speed, and depression on cognitive function in 755 non-demented community-dwelling elderly individuals. Cogn. Behav. Neurol..

[63] Blair M, Gill S, Gutmanis I, Smolewska K, Warriner E, Morrow SA (2016). The mediating role of processing speed in the relationship between depressive symptoms and cognitive function in multiple sclerosis. J. Clin. Exp. Neuropsychol..

[64] Posselt J, Klawunn R, Dierks M-L (2023). Digital health intervention prescriptions for people with depressive disorders: results of a qualitative study. Z. für. Allgemeinmedizin..

[65] Tagay S, Herpertz S, Langkafel M, Erim Y, Bockisch A, Senf W, Gorges R (2006). Health-related quality of life, depression and anxiety in thyroid cancer patients. Qual. Life Res..

[66] C. O’Neill, M. Carlson, C. Rowe, E. Fradgley, C. Paul, Hearing the voices of Australian thyroid cancer survivors: qualitative thematic analysis of semistructured interviews identifies unmet support needs. Thyroid 33, 1455–1464 (2023). 10.1089/thy.2023.0080 10.1089/thy.2023.0080 PMC10734898 37335225

